# *Plasmodium falciparum* encodes a conserved active inhibitor-2 for Protein Phosphatase type 1: perspectives for novel anti-plasmodial therapy

**DOI:** 10.1186/1741-7007-11-80

**Published:** 2013-07-09

**Authors:** Aline Fréville, Katia Cailliau-Maggio, Christine Pierrot, Géraldine Tellier, Hadidjatou Kalamou, Sophia Lafitte, Alain Martoriati, Raymond J Pierce, Jean-François Bodart, Jamal Khalife

**Affiliations:** 1 Center for Infection and Immunity of Lille, Inserm U1019-CNRS UMR 8204, University of Lille Nord de France, Institut Pasteur de Lille, 1 Rue du Professeur Calmette, 59019 Lille, Cedex, France; 2 EA4479, IFR147, Laboratoire de Régulation des Signaux de Division, SN3, Université des Sciences et Technologies de Lille, 59655 Villeneuve d’Ascq, France

**Keywords:** PP1, Plasmodium, RVXF motifs, Inhibitor-2, G2/M cell cycle

## Abstract

**Background:**

It is clear that the coordinated and reciprocal actions of kinases and phosphatases are fundamental in the regulation of development and growth of the malaria parasite. Protein Phosphatase type 1 is a key enzyme playing diverse and essential roles in cell survival. Its dephosphorylation activity/specificity is governed by the interaction of its catalytic subunit (PP1c) with regulatory proteins. Among these, inhibitor-2 (I2) is one of the most evolutionarily ancient PP1 regulators. *In vivo* studies in various organisms revealed a defect in chromosome segregation and cell cycle progression when the function of I2 is blocked.

**Results:**

In this report, we present evidence that *Plasmodium falciparum*, the causative agent of the most deadly form of malaria, expresses a structural homolog of mammalian I2, named PfI2. Biochemical, *in vitro* and *in vivo* studies revealed that PfI2 binds PP1 and inhibits its activity. We further showed that the motifs ^12^KTISW^16^ and ^102^HYNE^105^ are critical for PfI2 inhibitory activity. Functional studies using the *Xenopus* oocyte model revealed that PfI2 is able to overcome the G2/M cell cycle checkpoint by inducing germinal vesicle breakdown. Genetic manipulations in *P. falciparum* suggest an essential role of PfI2 as no viable mutants with a disrupted *PfI2* gene were detectable. Additionally, peptides derived from PfI2 and competing with RVxF binding sites in PP1 exhibit anti-plasmodial activity against blood stage parasites *in vitro*.

**Conclusions:**

Taken together, our data suggest that the PfI2 protein could play a role in the regulation of the *P. falciparum* cell cycle through its PfPP1 phosphatase regulatory activity. Structure-activity studies of this regulator led to the identification of peptides with anti-plasmodial activity against blood stage parasites *in vitro* suggesting that PP1c-regulator interactions could be a novel means to control malaria.

## Background

During its life cycle, *Plasmodium falciparum*, the deadliest malaria parasite for humans, undergoes several successive rounds of mitosis to amplify parasite populations and consequently to increase the rate of its transmission. Among the essential actors in the growth and division of the parasite are kinases and phosphatases. Indeed, initial experiments using inhibitors of these enzymes, such as staurosporine and okadaic acid drastically inhibited parasite growth *in vitro*[1-3]. Subsequently, the identification of kinases and phosphatases and their central functions in *P. falciparum*[4-9] demonstrated that phosphorylation and dephosphorylation represent a key post-translational modification regulating the activities of a variety of proteins. The former process is supported by a recent high-throughput phosphoproteomic studies of blood stage parasites that identified around 7000 phosphorylation sites on ~ 28% of proteins [4,10-12]. Of note is that the profile of the reported phosphoproteome reflects the global status of proteins resulting from a balance between endogenous kinase and phosphatase activities. *In vivo* studies, knocking down kinases in *Plasmodium*, and high-throughput screening of several thousand small chemical kinase inhibitors against blood stage parasites confirmed kinases as important drug targets [13-16].

Among the phosphatases, PP1 has been identified in *P. falciparum* (PfPP1c) and it accounts for the major phosphatase activity in total parasite extracts [1,17,18]. The use of potent inhibitors of phosphatases showed that *P. falciparum* predominantly expressed PP1-like activity which appears to control parasite growth and seems to be involved in the release of infectious merozoites [19,20]. In the past decade, a vast body of research has provided converging evidence that the key mechanism of the mode of action of the PP1c subunit resides in the presence of interacting regulators that direct the proper functions of this phosphatase (i.e. localization, specificity and the level of activity) [21-23]. At present, there are about 200 PP1 interacting proteins among which about 100 have been identified as regulatory subunits of PP1c [24-26]. The majority of regulators that inhibit the phosphatase activity interact with PP1c through an amino acid sequence present in the regulator and designated as the ‘RVxF’ motif. The consensus sequence [R/K]X0-1[V/I](p)[F/W], where X can be any amino acid and (p) any residue except proline, has been defined as a canonical PP1-binding site [27]. With respect to the endogenous regulators of PP1 and in comparison to other organisms, very few have so far been identified in *P. falciparum,* although we previously reported the identification of two regulators, PfLRR1 and Pf inhibitor-3 [28,29]. Characterization studies have shown that both regulators interact with PfPP1 and are present in the nucleus of blood stage parasites. Functional assays revealed that PfLRR1 dramatically decreased PfPP1 activity, like its homologues in other organisms [28]. Unexpectedly, PfI3 strongly increased PfPP1 activity *in vitro* and was unable to rescue yeast deleted for the expression of its ortholog. These data suggest that these regulators control PP1 activity in the nucleus and underscore the need for a better understanding of the function of PP1 regulators in each species [29].

A database search with Inhibitor-1 (I1) and Inhibitor-2 (I2), known to be powerful regulators of PP1c, identified one open reading frame in the *P. falciparum* genome (PlasmoDB gene identifier: PF3D7_0320000) encoding a potential protein with identity to known I2. Inhibitor-2 is a thermo- and acid-stable regulator initially purified from rabbit skeletal muscle and is conserved among all eukaryotes [30,31]. The potency of the inhibition by recombinant I2 of different species measured in parallel seems to be species specific in terms of inhibitory effect [32]. It is interesting to note that the peptide sequences of I2 orthologs vary in length, from 164 amino acids in plants up to 205 amino acids in humans. This may account for specificities mentioned above. The comparison of I2 sequences of different species along with *in vitro* functional studies revealed that two main regions participate in the interaction with PP1c and the inhibition of its activity: one binding region containing a KSQKW motif suggested to fulfill the role of the canonical RVxF motif and a second region containing a HYNE motif [33,34]. In addition, a third region present in the N-terminal moiety of human I2 and containing a KGILK motif has also been shown to be involved in the interaction with PP1c [34-36]. The resolution of the rodent PP1c-I2 crystal structure confirmed the implication of these regions in the binding process [37].

*In vivo*, the overexpression of GLC8 (a yeast I2 homologue) or the knockdown of human I2 by RNA interference showed its direct role in the cell cycle [38,39]. For instance, human I2 knockdown produced multinucleated cells during anaphase and blocked cytokinesis [39]. Moreover, exploration of the role of I2 in *Drosophila* development evidenced that an I2 loss-of-function in mothers leads to a dramatic reduction in the viability of progeny as measured by a decrease in embryonic hatch rates and larval lethality. However, I2 gain-of-function by transgenic expression of I2 in mutant mothers reversed this effect [40]. Altogether, these observations indicate that I2 plays a critical role in achieving successful mitosis and it is apparent that interfering with I2 functions represents an attractive approach for pharmacological intervention. Here, we report the structure-function relationship of inhibitor-2 of *P. falciparum* (PfI2) and explore its role and the means to affect its function in the parasite.

## Results

### Cloning and bioinformatics analysis of Pf Inhibitor-2

**Figure 1:**
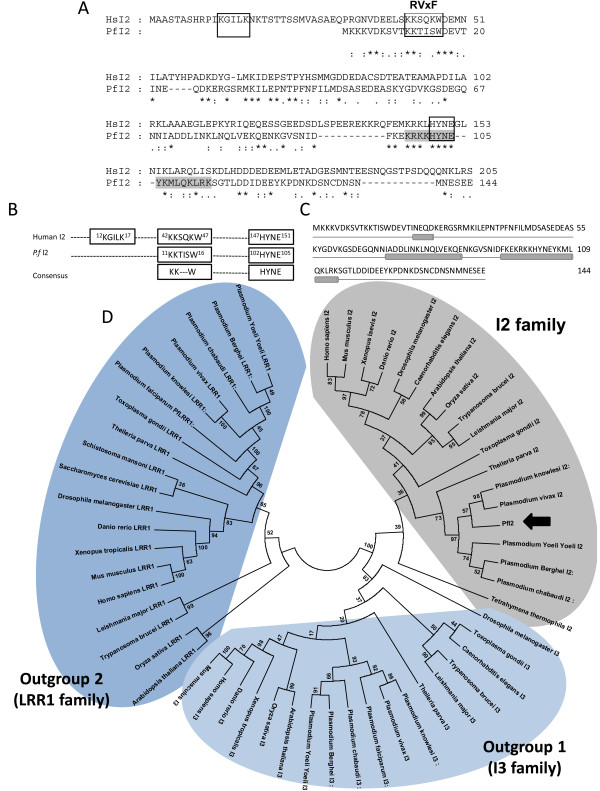
**Bioinformatic analysis. A.** Comparison between the deduced amino acid sequence of *P. falciparum* inhibitor 2 (PfI2) and Human inhibitor 2. (PlasmoDB gene identifier: PF3D7_0320000) was aligned with the Human Inhibitor 2 (HuI2 (Genbank accession number: NM_006241) homologous using the ClustalW algorithm and was manually corrected. The identical residues are shown by a star. Amino acids are numbered at the right of the sequence. The first box contains the “KGILK” motif, the second the “RvXF” motif and the third, the “HYNE” motif. The amino acids involved in the putative nuclear localization signal are shown in light gray. **B.** Known interaction sequences between Human I2 and PP1 were compared to PfI2 sequences. **C.** Predicted PfI2 structure. The helices are represented by a cylinder, the coils are represented by a line. **D.** Phylogenic tree of the Inhibitor 2 family. A maximum likehood tree was generated from the 21 inhibitor-2 sequences using MEGA5 under the JTT + G + I model with 100 bootstrap repetitions. Outgroups are formed by inhibitor 3 orthologs (I3, outgroup 1) and sds22 like proteins (LRR1, outgroup 2).

Analysis of PlasmoDB [41] using the human Inhibitor-2 sequence (CAA55475) identified a gene (gene identifier PF3D7_0320000) encoding a potential *P. falciparum* Inhibitor-2 homolog (PfI2). To establish the identity of the *PfI2* sequence, we determined the nucleotide sequence by RT-PCR using cDNA from total RNA of blood stage parasites and primers specified in Additional file 1: Table S1. The amplification showed a PCR product of the predicted size, confirming its transcription and the microarray data available in PlasmoDB. A walking approach on cDNA from the untranslated 5′ and 3′ ends allowed the validation of the start and stop codons respectively. The deduced amino acid sequence of the open reading frame corresponds to a protein containing 144 amino acids, indicating that PfI2 has the shortest amino acid sequence among I2 homologs (Additional file 2: Figure S1). Sequence alignment combined with visual inspection of PfI2 showed an overall identity of 28% and 34% identity between amino acids at positions 5 to 105 of PfI2 when compared to human I2 (Figure 1A). The use of PSORTII software [42] revealed a putative nuclear localization signal (sequence position ^98^KRKKHYNEYKMLQKLRK^114^) (Figure 1A). The PfI2 protein contains two peptides KTISW and KHYNE that fit perfectly to the [R/K]X[V/I]X[F/W] or RVxF motif and [K/R]HYNE motifs responsible for binding to PP1c (Figure 1B). However, 2 main differences were observed between PfI2 and human I2 proteins. First, the KGILK sequence, found in human I2 and shown to be required for interaction with PP1c [34-36] is not present in the PfI2 sequence and second, the KSQKW sequence of human I2 contains a Q residue instead of V or I of the RVxF consensus sequence (Figure 1A,B). The analysis of PfI2 using protein secondary structure prediction software PsiPred [43,44] predicted that the RVxF motif is a part of an unstructured region, while the HYNE motif is within an α-helix occurring between positions 70 and 120 (Figure 1C). This structure is in agreement with that identified in mammalian I2 [37]. This analysis is in accordance with the structure prediction presented in PlasmoDB [41].

A maximum likehood phylogenetic tree was generated under the JTT + I + G model with the support of two outgroups composed of two well described PP1 regulators: Inhibitor 3 (I3, outgroup 1) and LRR1 (outgroup 2) [28,29,45,46]. In this tree (Figure 1D) PfI2 segregates with orthologues from other *Plasmodium* species as well as the apicomplexan *Theileria* parva, but within the I2 family on a well-supported branch (bootstrap analysis) separate from the I3 family. This analysis clearly identifies PfI2 as a PP1c inhibitor 2 family member.

### Expression of PfI2 protein by *P. falciparum* and localization studies

**Figure 2:**
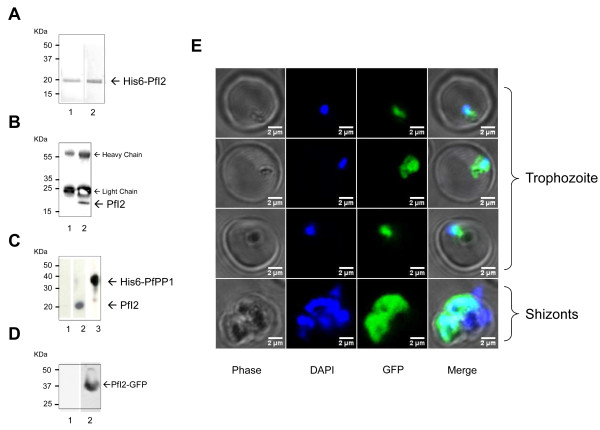
**Expression and localization of the *PfI2 *gene product by *P. falciparum*. A. **Purified His6-PfI2 separated by 15% SDS-PAGE and blotted onto nitrocellulose (Red Ponceau staining, lane 1) and revealed with mAb anti-His (lane 2) showed a single band at ~ 20 kDa, indicating an anomalous electrophoretic migration of PfI2 (expected size 16.7 kDa). The identity of the purified recombinant PfI2 was further confirmed by MALDI-TOF mass spectrometry. **B.** Immunoprecipitation of native PfI2 with anti-PfI2 polyclonal antibodies from *Plasmodium falciparum* extracts, followed by an immunoblot analysis with preimmune serum (lane 1) or with anti-PfI2 antisera (lane 2). **C.** Detection of PfI2 in total proteins extracted from asynchronous cultures of *P. falciparum* using PfPP1 column. Total protein extracts (10 mg) pre-cleared on Ni-NTA sepharose beads were incubated overnight with His6-PfPP1 affinity Ni-NTA column as described in Methods. The blots were probed with preimmune serum (lane 1), anti-PfI2 (lane 2) or with anti-His mAb antibodies (lane 3). The blots were revealed as described in Methods. **D.** Immunoblot analysis of pARL2-PfI2-GFP transfected *P. falciparum*. Protein extracted from wild-type parasites (lane 1) or from transfected parasites (lane 2) were subjected to western-blotting and probed with anti-GFP mAb antibodies. **E.** Expression and localization of PfI2-GFP throughout the erythrocytic cell cycle of *P. falciparum.* Parasites were transfected with pARL2-PfI2-GFP construct as described in Methods and live transfectants were analysed by fluorescence microscopy.

To investigate the expression of PfI2 by *P. falciparum*, polyclonal antibodies against the recombinant PfI2 protein were raised. As presented in Figure 2A lane 1 (Red Ponceau staining), the recombinant protein whose amino acid sequence was confirmed by MALDI-TOF mass spectrometry, migrated at about 20 kDa, in agreement with the anomalous electrophoretic behavior of inhibitors of the PP1 family; the expected molecular weight of endogenous PfI2 is 16.7 KDa. Although these antibodies recognized the recombinant protein (Figure 2A, lane 2), they were unable to react with any bands in total extracts of asynchronous blood stage parasites. In order to detect endogenous PfI2, we carried out immunoprecipitation experiments with anti-PfI2 sera or pre-bleed sera with total parasite extracts. Immunoblots with anti-PfI2 antibodies showed the presence of a band at 20 kDa in the immunoprecipitates with anti-PfI2 (Figure 2B, lane 2), while the pre-bleed serum detected no specific band (Figure 2B, lane1). To confirm this expression and to examine whether native PfI2 could be among the partners of PfPP1, we performed affinity purification from total parasite extracts using recombinant His-tagged PfPP1 retained on Ni-NTA agarose beads. As depicted in Figure 2C, immunoblot analysis of eluates with anti-PfI2 antibodies (lane 2) reacted with one band at 20 kDa, corresponding to the migration of the recombinant PfI2 protein. Lane 3 confirmed the presence of His-tagged PfPP1 by the use of mAb anti-His antibody.

To accurately follow up the distribution of PfI2 during the intraerythrocytic development cycle, we examined 3D7 parasites transfected with a pARL2 construct mediating the episomal expression of full-length GFP-fused *PfI2*. The use of this vector by Kuhn *et al.* showed that the trafficking was attributable to the nature of the protein expressed rather than to the *PfCRT* promoter used [47]. Using a mAb anti-GFP antibody, immunoblot analysis of a total extract of blood stage parasites expressing PfI2-GFP revealed the presence of a specific band at 37 kDa, which is the expected molecular mass of PfI2-GFP (Figure 2D, lane 2). This demonstrates the integrity of the fused protein in transfected parasites. Examination of live parasites showed that the signal was confined within the parasite where the distribution seemed to be nucleo-cytoplasmic (Figure 2E), as the fluorescence partially overlapped DNA staining. The distribution appeared to be diffuse in the late parasite stages with most staining in the nucleus. These results are in accordance with previous localization studies carried out on mammalian [48,49] or plant [50] cells showing a nucleo-cytoplasmic localization with an accumulation in the nucleus when human cells progressed into S phase [48]. The PfI2-GFP signal was completely absent from the digestive food vacuole (Figure 2E).

### Genetic manipulation of PfI2

**Figure 3:**
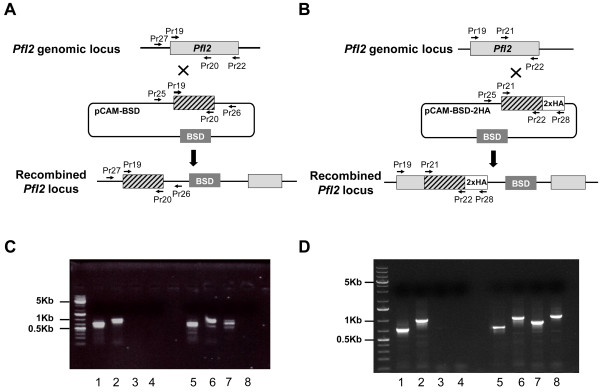
**Targeted gene disruption and HA-tagging of the PfI2 locus. A.** Disruption of PfI2 by knock-out strategy using single homologous recombination. The pCAM-BSD construct, the blasticidin-resistance cassette (BSD), the location of the primers (Additional file 1: Table S1) used for PCR analysis and the locus resulting from integration are indicated. **B.** Insertion of an HA epitope tag at the PfI2 C terminus (Knock-in strategy). **C.** Analysis of pCAM-BSD-PfI2 transfected 3D7 culture by PCR (Knock-out strategy). Lanes 1–4 correspond to DNA extracted from wild type parasites, lanes 5–8 to DNA extracted from transfected parasites. Lanes 1 and 5 represent the detection of a portion of wild type locus (Pr19 and Pr20); lanes 2 and 6, the detection of wild type locus (Pr19 and Pr22); lanes 3 and 7 show the detection of episomal DNA (Pr25 and Pr26) and lanes 4 and 8 show the detection of the integration at the 5′end of the insert (Pr27 and Pr26). The absence of PCR product amplification using genomic DNA prepared from transfected parasite culture (Pr27 and Pr26) indicated the lack of homologous recombination (lane 8). **D.** PCR analysis of pCAM-PfI2-2HA transfected 3D7 culture (Knock-in strategy)). Lanes 1–4: DNA extracted from wild type parasites; lanes 5–8: DNA extracted from transfected parasites. Lanes 1 and 5: detection of a portion of wild type locus (Pr21 and Pr22); lanes 2 and 6: detection of wild type locus (Pr19 and Pr22); lanes 3 and 7: detection of episomal DNA (Pr25 and Pr28) and lanes 4 and 8: detection of the integration at the 3′end of the insert (Pr19 and Pr28). The amplification of a PCR product at ~ 1000 pb using genomic DNA prepared from transfected parasite culture (Pr19 and Pr28) indicated the homologous recombination and integration of the 2-HA construct in endogenous PfI2 (lane 8).

To study whether the lack of PfI2 expression could affect the *Plasmodium* blood stage life cycle, attempts to disrupt the *PfI2* gene using the pCAM vector system were carried out. We transfected blood ring stage parasites of the 3D7 strain with a pCAM-BSD-PfI2 construct containing a 5′ fragment derived from the genomic *PfI2* sequence and the BSD gene conferring resistance to blasticidin (Figure 3A). The presence of this construct in transfected parasites was checked by a plasmid rescue approach as previously described [29] (data not shown). From two independent transfection experiments, the analysis of genomic DNA obtained from resistant stable parasites by PCR (from 2 months up to 9 months of culture under blasticidin pressure), with specific primers indicated in Additional file 1: Table S1, did not evidence the interruption of the *PfI2* gene (Figure 3C, lane 8). The wild type gene was still amplified in genomic DNA even after prolonged culture (> 9 months of culture) and the plasmid remained episomal.

The absence of knock-out parasites could be attributed either to the essentiality of PfI2 or to the lack of accessibility of *PfI2* to genetic manipulations. To exclude the latter hypothesis and to check the accessibility for recombination of the *PfI2* locus, we introduced a targeted modification in the locus without loss-of-function. To this end, 3D7 ring stage parasites were transfected with a plasmid containing the 3′ end of the *PfI2* coding region fused to the hemagglutinin sequence (Figure 3B). Genotype analysis by PCR, using one specific primer of *PfI2* derived from the upstream region of the construct *PfI2-HA* and a primer corresponding to the hemagglutinin sequence showed the presence of a specific PCR product at the expected size, indicating the correct integration of *PfI2-HA* into the locus (Figure 3D, lane 8). Taken together, these data suggest the essentiality of PfI2 for the survival of blood stage parasites.

### Effect of PfI2 on Phosphatase activity of PfPP1

**Figure 4:**
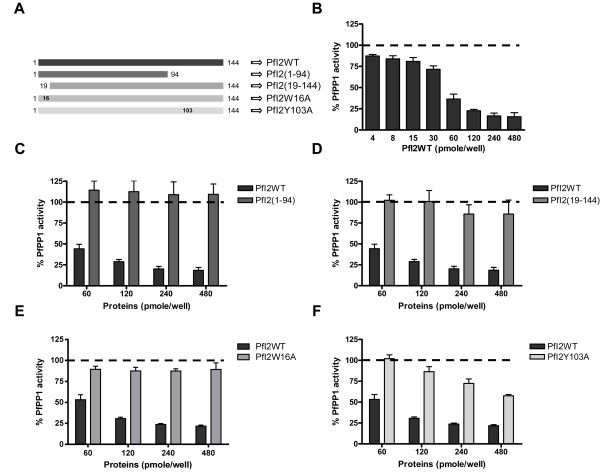
**Effect of PfI2 on PfPP1 phosphatase activity. A.** Scheme of His-tagged recombinant wild-type, deleted and mutated PfI2 proteins used in this study. Recombinant PfPP1 was pre-incubated for 30 min at 37°C with different concentrations of PfI2WT **(B)**, PfI2(1–94) **(C)**, PfI2(19–144) **(D)**, PfI2W16A **(E)** and PfI2Y103A **(F)** proteins before the addition of pNPP. Results presented as% of relative increase or decrease are means ± SEM of four independent experiments performed in duplicate.

Next, we assayed PfI2 for its potential capacity to regulate PfPP1 activity. As previously described, PfPP1 produced as a recombinant protein dephosphorylates the pNPP substrate, is sensitive to known PP1 inhibitors and its activity is Mn2^+^-dependent [28]. Using a concentration of recombinant PfPP1 within a range producing linear release of phosphate, the effect of wild-type recombinant (PfI2WT), deleted or mutant recombinant PfI2 proteins was evaluated as described in Methods. Deleted or mutated PfI2 versions presented in Figure 4A were produced as recombinant proteins and used in the functional assay. Results showed a strong decrease (up to 80% inhibition) in the phosphatase activity when PfPP1 was pre-incubated with PfI2WT (Figure 4B). As PfI2 contains the 2 main motifs, ^12^KTISW^16^ (RVxF consensus motif) and ^102^HYNE^105^, known to be essential for the function of Inhibitor-2, we explored the impact of these motifs on PfI2 function in terms of PP1 inhibition. The deletion of either the Nt (PfI2(19–144)) or Ct (PfI2(1–94)) portion containing the RVxF and HYNE motifs of PfI2 respectively abolished its inhibitory function almost completely (Figure 4C, 4D). When the PfI2W16A mutant protein was tested, we observed that this mutation led to an almost complete loss of function of PfI2, whatever the concentration of PfI2W16A used (Figure 4E). The PfPP1 activity detected was identical to the control. In the case of the PfI2Y103A mutant protein, a loss of function was observed at the lowest concentration, however, at higher concentrations of PfI2Y103A a decrease of up to ~50% of PfPP1 activity was observed (Figure 4F), suggesting that this mutation only partially affected the function of PfI2. These data suggest that the RVxF motif is the major contributor for the function of PfI2.

### Study of PfI2-PfPP1 interaction and mapping of binding motifs

**Figure 5:**
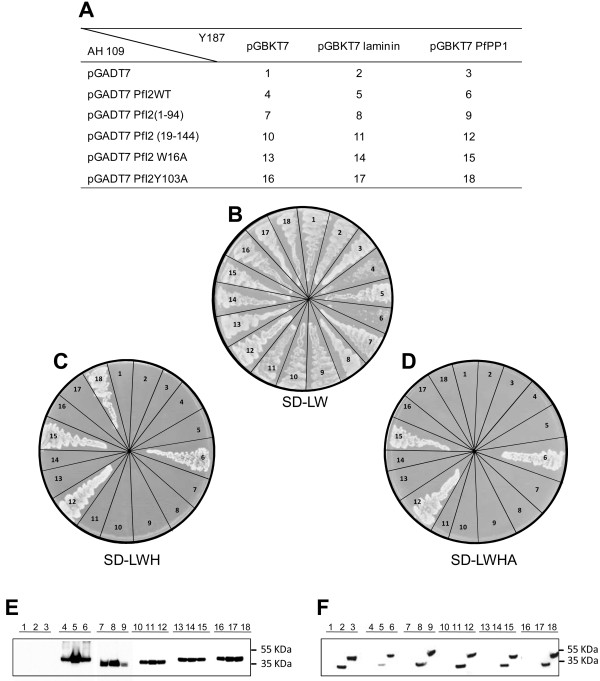
**Study of PfI2-PfPP1 interaction and mapping of binding motifs in yeast two hybrid system.** pGBKT7, pGBKT7-lam, pGBKT7-PfPP1 constructs were inserted into mat α (Y187) yeast and pGADT7, pGADT7-PfI2, pGADT7-PfI2(1–94), pGADT7-PfI2(19–144), pGADT7-PfI2W16A and pGADT7-PfI2Y103A constructs into mat a (AH109) yeast. These transformations were followed by mating **(A)**. Yeast diploids were checked on SD-LW plates **(B)** and interactions were identified by growth on SD-LWH **(C)** or SD-LWHA **(D)**. Western-blot analysis of extracts prepared from yeast diploids revealed with mAb anti-Gal4-AD **(E)** or mAb anti-Gal4-BD **(F)** showing the expected expression of the Gal4-AD tagged PfI2 proteins (PfI2WT: lanes 4,5,6; PfI2(1–94): lanes 7,8,9; PfI2(19–144): lanes 10,11,12; PfI2W16A: lanes 13,14,15 and PfI2Y103A: lanes 16,17,18) and Gal4-BD tagged laminin : lanes 2,5,8,11,14,17 or Gal4-BD tagged PfPP1: lanes 3,6,9,12,15,18.

The loss of function of deleted/mutated PfI2 observed above may be related to its failure to interact with PfPP1. Hence, the binding capacity of wild-type, deleted and mutated PfI2 with PfPP1 was assessed using the yeast two-hybrid system. The interaction between PfPP1-Gal4-BD and PfI2-Gal4-AD can be evidenced by growing diploid strains on SD media lacking Leucine, Tryptophan, Histidine (SD-LWH) or SD-LWHA (A for Adenine). Mating assays between different strains are summarized in Figure 5A, including those with control constructs. All mated strains were shown to be able to grow on SD-LW (Figure 5B), indicating that they contained the PfI2 and PfPP1 constructs. Western blot analysis showed the expression of tagged PfPP1 (anti-BD-Gal4 antibody) (Figure 5F) and the expression of PfI2 (anti-AD-Gal4 antibody) (Figure 5E). The diploid strains containing PfPP1 and PfI2WT or deleted/mutated PfI2 (PfI2(19–144), PfI2W16A or PfI2Y103A) showed similar growth of these strains on SD-LWH media (Figure 5C), indicative of an interaction with PfPP1. However, the diploid strains containing PfPP1 and PfI2(1–94) or control plasmids were unable to grow. When stringent culture conditions were applied using SD-LWHA medium, the strains containing PfPP1-PfI2WT, PfPP1-PfI2(19–144) or PfPP1-PfI2W16A were still able to grow while the strain containing PfPP1-PfI2Y103A lost its capacity for growth (Figure 5D), suggesting a role for Y103 in the stability of the interaction. Taken together, these results suggest that the loss of function of most deleted or single mutated PfI2 proteins is not due to a loss of interaction with PfPP1.

### Initiation of G2/M in *Xenopus* oocytes by PfI2

**Figure 6:**
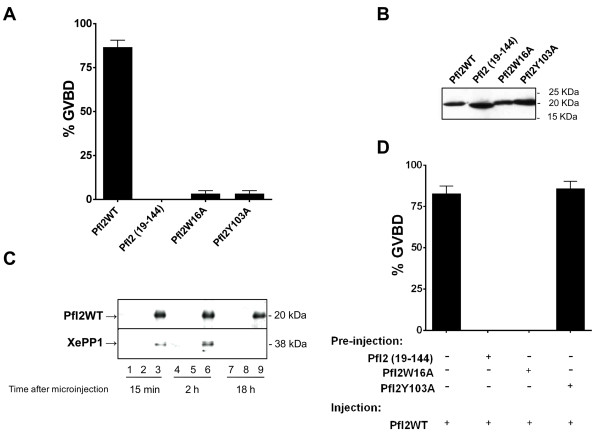
**Initiation of G2/M transition in *Xenopus *oocytes by PfI2.** Appearance of GVBD was monitored for 15 h after injection, and values are presented as percentages. Each experiment was performed using a set of 20 oocytes and was repeated at least three times. **A.** Percentage of GVBD induced with 100 ng of PfI2WT, PfI2(19–144), PfI2W16A or PfI2Y103A recombinant proteins. **B.** Immunoblot analysis of extracts prepared from injected oocytes revealed with mAb anti-His showing the presence of each recombinant protein after microinjection **C.** Interaction of PfI2 with *Xenopus* PP1. Immunoblot analysis using specific anti-XePP1 antibodies of naïve (lanes 1,4,7) or PfI2WT injected oocytes extracts after co-immunoprecipitation with anti-His mAb (lanes 3,6,9) (or anti-rabbit used as control (lanes 2,5,8)) revealed the presence of XePP1 in the complex (lanes 3,6). **D.** Percentage of GVBD induced by the pre-injection of PfI2(19–144), PfI2W16A or PfI2Y103A (100 ng) 2 hours before PfI2WT injection (100 ng). PfI2WT injection was used as a positive control.

The partial conservation in PfI2 of two PP1 binding motifs likely suggests a capacity to interact with other PP1 (highly conserved between species >80% identity) and to exert a potential function. Previous studies reported that the inhibition of PP1 in *Xenopus* oocytes by anti-PP1 antibodies triggered G2/M transition measured by the appearance of Germinal Vesicle Break Down or GVBD [51]. Having established the inhibitory role of recombinant PfI2 on the phosphatase activity of PfPP1 *in vitro*, we followed up the induction of GVBD by microinjecting the wild or mutated His-tagged PfI2 proteins. Also, we evaluated the ability of Nt deleted PfI2 (PfI2(19–144)) to trigger G2/M transition as it is still able to bind PP1 in the absence of the RVxF motif. Results presented in Figure 6A indicated that PfI2WT was able to induce GVBD (~ 85%). Under the same conditions, PfI2(19–144), PfI2W16A or PfI2Y103A proteins were ineffective in inducing GVBD. The presence of each protein in microinjected oocytes was checked by immunoblots using anti-His mAb (Figure 6B). In parallel, it was essential to check whether PfI2WT can bind to *Xenopus* PP1 (XePP1). As shown in Figure 6C, the use of a specific PP1 antibody for immunoblot analysis of eluates co-immunoprecipitated with anti-His mAb revealed the presence of XePP1 in the complex (lane 3, lane 6). The complex PfI2WT-XePP1 was detected in *Xenopus* extracts 15 mn post micro-injection (lane 3).

The loss of functions of PfI2(19–144), PfI2W16A and PfI2Y103A, combined with the fact that they retain their capacity to bind to PfPP1, prompted us to examine their capacity to block the function of PfI2WT. For this, oocytes were pre-injected with the deleted or mutated PfI2 proteins, incubated for 2 hr and followed by the injection of PfI2WT. Results showed that PfI2(19–144) as well as PfI2W16A were able to completely abrogate the function of PfI2WT as no GVBD was observed (Figure 6D). However, PfI2Y103A did not inhibit the function of PfI2WT.

### Inhibition of PfI2 function by synthetic peptides

**Figure 7:**
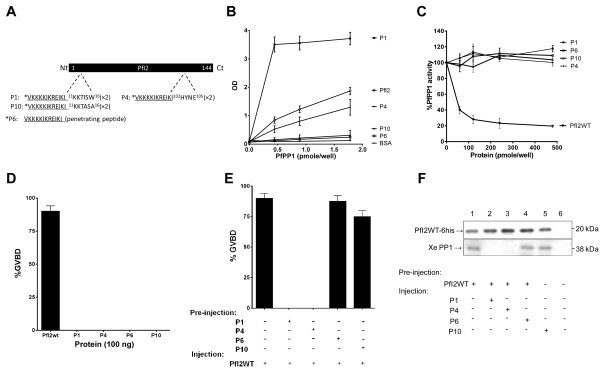
**Role of synthetic peptides derived from PfI2. A.** Schematic representation of PfI2 sequence and P1 and P4 peptides derived, respectively, from the RVxF (^11^KKTISW^16^) and HYNE (^102^HYNE^105^) domains. P6 (penetrating peptide) and P10 (P1 mutated version) were used as negative controls. **B.** Interaction studies of P1, P4, P6, P10 with His6-PfPP1 *in vitro* using ELISA based quantification of binding capacity. Increasing quantities of biotinylated His6-PfPP1 were added to wells coated with peptides (5 μg/well) or recombinant PfI2WT protein (5 μg/well) used as positive control. Results are representative of 4 independent experiments. **C.** Effect of P1, P4, P6 and P10 on PfPP1 phosphatase activity. Recombinant His6-PfPP1 at 27.03 nM (2.5 μg) was pre-incubated for 30 min at 37°C with different concentrations of peptides or PfI2WT (positive control) before the addition of pNPP. Results presented as % of relative increase or decrease are means ± SEM of four independent experiments performed in duplicate. **D.** Initiation of G2/M in *Xenopus* oocytes by peptides. Appearance of GVBD was monitored for 15 h after injection, and values are presented as percentages. Each experiment was performed using a set of 20 oocytes and was repeated at least three times. PfI2WT injection was used as positive control. **E.** Percentage of GVBD induced by the pre-injection of P1, P4, P6 or P10 (100 ng) 1 hour before PfI2WT injection (100 ng). PfI2WT injection was used as a positive control. **F.** Abolition of PfI2WT/XePP1 interaction after P1 and P4 peptides pre-injection. Extracts from oocytes pre-injected or not with peptides followed by PfI2WT injection were immunoprecipitated with anti-His mAb. Immunoblot analysis revealed with specific anti-XePP1 antibodies revealed the presence of XePP1 in the complex in P6 and P10 pre-injected extracts (lanes 4 and 5) and in non-pre-injected extracts (lane 1) but not in the P1 and P4 pre-injected extracts (lanes 2 and 3).

From the above results, it appears that W16 and Y103 of PfI2 are critical residues within the KTISW (RVxF) and HYNE motifs for binding/inhibition of PP1 with a stronger role for the former. In addition, mutated PfI2 blocked the function of the full-length PfI2WT. Consequently, we investigated whether synthetic peptides containing these motifs (Figure 7A and Additional file 3: Table S2) could bind to PP1 and inhibit the function of PfI2WT. Using ELISA based experiments we found that PfPP1 was able to bind specifically to peptides containing either the KTISW (RVxF) or HYNE motifs (Figure 7B). A higher capacity of PfPP1 to bind the KTISW-containing peptide (P1) compared to the HYNE-containing peptide (P4) was observed. Interestingly, in PfPP1 activity assays, and unlike PfI2WT, the synthetic peptides did not exhibit any capacity to inhibit PfPP1 activity (Figure 7C). The absence of any effect of peptides alone on PP1 activity was further confirmed *in vivo* as their microinjection into *Xenopus* oocytes did not induce GVBD (Figure 7D). Hence, we investigated whether synthetic peptides are able to block PfI2WT function as measured by GVBD induction. Oocytes were pre-injected with peptides before the injection of PfI2WT. Results presented in Figure 7E revealed that the microinjection of either KTISW-containing peptide (P1) or the HYNE-containing peptide (P4) almost completely abolished GVBD induction. Pre-injections of control peptides (P6 and P10) did not lead to any inhibition of PfI2WT-dependent GVBD. Immunoblot analysis of co-immunoprecipitates with anti-His mAb demonstrated that the pre-injections of P1 or P4 peptides prevented the binding of PfI2WT to XePP1 while the control peptides did not (Figure 7F).

### Effect of peptides competing with PfI2 on the growth of blood-stage *P. falciparum* parasites

**Figure 8:**
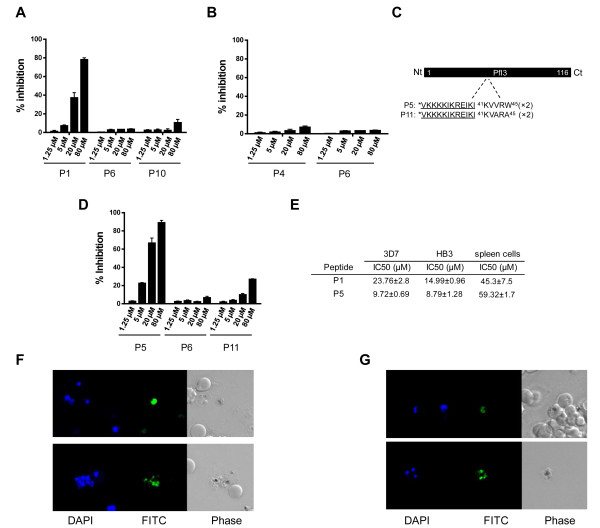
**Effect of synthetic peptides derived from PfI2 and PfI3 on blood *P. falciparum *parasite growth. A** and **B.** Inhibition of growth of 3D7 *P. falciparum* strain parasites by PfI2 derived peptides. Parasites were incubated with different concentrations of synthetic peptides for 72 hr in a 96-well microtiter plate. Parasitemia was quantified as described in Methods. P1 and P4 correspond to KTISW and HYNE motifs respectively. P6 (penetrating peptide alone) and P10 (mutated version of P1) were used as control peptides. **C.** Schematic representation of PfI3 sequence (Pf Inhibitor 3): P5 peptide corresponds to ^41^KVVRW^45^ (RVxF motif) and P11 is the mutated version of P5 used as control peptide. **D.** Inhibition of growth of *P. falciparum* 3D7 by synthetic peptides derived from PfI3. Results presented as% of parasite growth inhibition are means ± SEM of seven independent experiments performed in duplicate. **E.** IC50 values of P1 and P5 against 3D7 and HB3 *P. falciparum* strains and against stimulated primary mouse spleen cells. F. Selective penetration of FITC-labeled P1 **(F)** and P5 **(G)** peptides in *P. falciparum* 3D7. The left panel represents the DAPI staining, the middle panel represents the fluorescence signal and the right panel represents the bright field image*.*

The ability of synthetic peptides to block the effect of PfI2WT using the *Xenopus* model, combined with the observation suggesting that PfI2 is essential in *P. falciparum* blood-stage parasites, led us to evaluate the capacity of these peptides to inhibit the growth of *P. falciparum in vitro*. The synthetic peptides with repeated motifs of either the RVxF motif (KTISW) (P8) or the HYNE motif (P9) (Additional file 3: Table S2) did not show any effect on parasite growth (not shown) which could be due to very low or absence of peptide penetration. To improve and enhance peptide uptake, the penetrating peptide VKKKKIKREIKI (P6), previously shown to act as a non-toxic shuttle to deliver peptides to infected red blood cells [52] was coupled to the NH2 terminus of each repeated motif. As shown in Figure 8A, the peptide P1 containing the KTISW motif inhibited parasite growth in a dose dependent manner with an inhibition of ~80% at a concentration of 80 μM. Negative controls including peptides corresponding to the penetrating peptide alone (P6) or to the mutated peptide (P10) did not show specific inhibition. Regarding the peptide containing HYNE (P4), no growth inhibition of blood stage parasites was detectable (Figure 8B) (<10% at 80 μM) although it was able to block the function of PfI2WT in the oocyte model (Figure 7E).

To confirm the role of the RVxF competing peptide on *P. falciparum* growth, a second motif (KVVRW) derived from Pf Inhibitor-3, which we previously reported as the RVxF motif of this inhibitor [29], was evaluated under the same conditions. Results presented in Figure 8D indicate that the peptide containing the KVVRW sequence (P5) did potently reduce parasitemia (up to 90% of inhibition at a concentration of 80 μM), while the mutated corresponding peptide (P11) exhibited a drastically reduced capacity to inhibit parasite growth. As we observed a sigmoidal concentration effect relationship, IC50 values for the active peptides were calculated. Based on 7 independent experiments carried out on the 3D7 strain with 3 different batches of synthetic peptides, IC50s were 23.76 μM for KTISW (P1) and 9.72 μM for KVVRW (P5) containing peptides respectively (Figure 8E). When the effect of P1 and P5 peptides was tested on the HB3 strain (2 different batches), the IC50s were 14.99 μM and 8.79 μM respectively. Finally, the toxicity of these peptides was evaluated by their capacity to block the proliferation of stimulated mouse spleen cells *in vitro*. The calculated IC50 was 45.3 μM for the KTISW-containing peptide and 59.32 μM for the KVVRW-containing peptide (n = 3 independent experiments), showing a selectivity index of 2 to 6 fold for *P.falciparum* according to the peptide tested.

To further explore the uptake of active P1 and P5 peptides by blood parasite stages, FITC-labeled peptides were used. As shown in Figure 8F, FITC-P1 was only accumulated within free merozoites, while FITC-P5 penetrated infected red blood cells and concentrated within intracellular parasites (Figure 8G upper panel) as well as free merozoites (Figure 8G, lower panel). Uninfected red blood cells did not accumulate any FITC-peptide (Figure 8F, Figure 8G).

## Discussion

The *Pf Inhibitor2* gene (*PfI2*) encodes a protein of 144 amino acids related to the I2 proteins of different organisms, which are known to inhibit PP1c activity *in vitro*. Of the three central regions identified in the I2 protein as binding motifs to PP1, the KGILK, RVxF, and HYNE motifs, PfI2 contained only a consensus RVxF (^12^KTISW^16^) and the ^102^HYNE^105^ sequences. The lack of KGILK in PfI2 was supported by bioinformatics analysis indicating the absence of this sequence in all potential open reading frames upstream of the *PfI2* gene and was further confirmed by a 5′cDNA walking approach. The KGILK motif present in vertebrate I2 was found to be involved in the interaction with PP1 through the region of amino acids 50–59 in PP1c [37]. In addition, deletion of the N-terminal side of I2 containing this site and mutation of the first Lys or the Ile dramatically reduced the inhibition capacity of I2 (>500 fold decrease) [33-35]. These observations emphasize the importance of this site in the binding and activity of vertebrate I2, which represents a major difference compared with PfI2, which lacks this motif. Concerning the RVxF site, vertebrate I2 does not contain the canonical motif falling within the consensus sequence [R/K]X0-1[V/I]X0-1[F/W]. However, studies on the crystal structure of PP1c-I2 revealed that the sequence KSQKW, where the consensus Val/Ile residue is replaced by a Gln is docked in the PP1 groove, which usually binds the RVxF motif [37]. Structure-activity studies on the implication of KSQKW site showed that the mutation of Trp in mammalian I2 drastically reduced the inhibitory activity of I2 [33]. It is worth noting that almost all I2 proteins contain Gln at the position of Val/Ile. However, in *P. falciparum*, the I2 protein does contain an Ile in the RVxF motif, a second important dissimilarity between PfI2 and other I2 proteins. The comparison of PfI2 with putative I2 of *Toxoplasma gondii* or *Neospora canium* (TGGT1_114760, NCLIV_032710), revealed the presence of the consensus RVxF sequence where V/I is replaced by Leu, maintaining the hydrophobicity of the residue and suggesting its conservation within other Apicomplexa parasites. Studies on the third site of interaction, HYNE, have shown that the His and Tyr residues are important in the interaction with PP1c and it has been proposed that this motif functions as a ‘degenerate’ RVxF motif [33,53]. More recent studies clearly showed that the region containing the HYNE motif interacts directly with the active site of PP1c (involving ^272^Tyr and ^96^Arg residues of PP1c) with a major contribution of His and Tyr residues [33,37]. This excludes completely the possibility of a competition of binding to PP1c between the RVxF and HYNE motifs and suggests that the His and Tyr residues of I2 promote the displacement of the catalytic metal ion. In the PfI2 protein, these two residues are conserved.

Among the three binding sites of I2, the best-identified and most widely found in PP1 partners is the [R/K]X0-1[V/I]X0-1[F/W] consensus motif, which corresponds to KTISW in PfI2. The presence of RVxF in about 25-30% of eukaryotic proteins is not a sufficient indicator in itself to classify a protein as a PP1c regulator [54]. These observations, together with the fact that PfI2 is the shortest I2 protein identified so far (144 amino acids for PfI2 versus 164–205 for other I2), the absence of one binding site (KGILK) and the fundamental difference in the RVxF motif (KTISW) raised the question of the capacity of PfI2 to bind and to regulate PfPP1. Using wild-type recombinant proteins, we showed that labeled PfPP1 was able to bind to PfI2 and *vice versa*. This was further validated by the use of a yeast two-hybrid system that confirmed the interaction of wild-type PfI2 with PfPP1c and suggested that it was strong since the mated PfI2 and PfPP1 yeast strains were able to grow under stringent conditions (SD-LWHA medium). In order to explore the contribution of PfI2 RVxF and HYNE motifs for the interaction with PfPP1, two types of constructions were used, one deleted for the Nt moiety of PfI2 and the other with a single mutation in the RVxF motif. Binding was unaffected on SD-LWH medium, whatever the construction tested and only one strain, carrying the PfI2 Y103A, mutant was unable to grow under the most stringent conditions (SD-LWHA medium). These observations show that there is no one, major site of interaction in PfI2 unlike Pf Inhibitor-3 (PfI3), for which we showed that the mutation of ^16^ W (localized within the RVxF domain of PfI3) completely abolished its binding/function [29]. PfI3 exhibits a totally disorganized structure and seems to bind first to PfPP1 via the RVxF groove and folds afterwards to accomplish its function [29]. Regarding I2, previous studies suggested a major role for the RVxF motif along with secondary binding sites which should be intrinsically structured for efficient binding to PP1c [33-35]. PfI2 secondary structure analysis predicted that the RVxF motif is a part of an unstructured region, while the HYNE is within an α-helix. The role of this structure in PfI2-PfPP1c interaction was substantiated by the lack of binding of PfI2 deleted for the region containing the α-helix (PfI2 (1–94)). In the case of mutated PfI2, the yeast two-hybrid method supported a role for ^103^Tyr (localized within the HYNE domain of PfI2) in the stabilization of PfI2-PfPP1 binding under stringent culture conditions.

It has been shown that most I2 proteins are able to drastically decrease PP1c activity towards different non-specific substrates such as Phosphorylase A and pNPP [34,35,38,50]. As expected, the addition of PfI2 in the nanomolar range significantly decreased PfPP1 activity up to 80%. To investigate the impact of KTISW (RVxF) and HYNE motifs on PfI2 regulatory activity we used deleted or mutated recombinant proteins. The contribution of the RVxF motif (KTISW) is key to the function of PfI2 as both Nt deleted PfI2 (PfI2(19–144)) and mutated PfI2 (PfI2W16A) were unable to inhibit PfPP1 activity, whereas the involvement of the HYNE domain seems to be less important. Thus, although the PfI2W16A mutant is still able to bind to PfPP1, ^12^KTISW^16^ is a vital and a primary site for the inhibitory activity of PfI2. To further evaluate the inhibitory activity of PfI2 and the role of the two motifs, we took advantage of the *Xenopus* model where oocytes are physiologically arrested in G2/M prophase I [55,56]. The injection of *Xenopus* I2 (spanning 188 residues and containing the KGILK, KSQKW and HYNE motifs) or anti PP1 antibodies into oocytes induced germinal vesicle breakdown or GVBD [51,57]. *Plasmodium* I2 is able to substitute for the Xenopus orthologue in this system since the microinjection of PfI2WT into oocytes promoted the progression to M phase, inducing GVBD and co-immunoprecipitation experiments confirmed the interaction of PfI2 with *Xenopus* PP1c. This confirmed that PfI2 can function in cells without the need for the KGILK site and are in accordance with previous studies that showed the involvement of *Xenopus* I2 in the G2/M transition in acellular extracts [57] or the implication of Glc8 (yeast inhibitor 2) in the cell cycle [38-40]. Deletion, mutation or RNA interference studies carried out on inhibitor 2 have demonstrated its implication in the cell cycle, chromosome segregation and embryogenic development [38,39,57]. In the case of PfI2, when deleted PfI2 (PfI2(19–144)) lacking ^12^KTISW^16^ or mutated PfI2 (PfI2W16A or PfI2Y103A) were microinjected, no GVBD was observed, demonstrating the importance of both PfPP1 binding sites in the functional capacity of PfI2. Since the PfI2 mutated proteins are able to bind PP1 but unable to inhibit its function we sought to determine whether the pre-injection of deleted or mutated PfI2 proteins may block the role of wild PfI2. The pre-injection of either PfI2(19–144) or PfI2W16A were able to block the induction of GVBD while PfI2Y103A did not. One explanation for these observations is that the HYNE-dependent binding is critical as the injection of PfI2WT is able to displace this mutated protein and to induce GVBD. When the HYNE site is not mutated the binding of PfI2 is sufficiently stable to prevent its displacement.

Closer examination of the PfI2 peptide sequence revealed the presence of a consensus PXTP motif (^37^PNTP^40^), also present in other I2, in which the phosphorylation of the T within this site abrogated the function of I2 [32,57]. In PfI2, the replacement of T by D (mimicking phosphorylation) did not impact either the binding or the function of PfI2 (not shown), tending to exclude the phospho-regulation of I2 at this site. These data are in agreement with the recent *P. falciparum* phosphoproteome characterization showing the phosphorylation of PfI2 at positions T^13^, S^48^, S^50^, S^115^, T^117^ and S^142^[4], but not at T^39^ within the PXTP motif. The assessment of the impact of PfI2 phosphorylation will await further investigations on these phosphorylated residues as well as the “T” within the PXTP motif. At this stage, it is important to mention that, beside the capacity to interact with PP1c, human I2 has been shown to participate in a direct kinase-dependent signaling network. It was found that I2 was able to bind and to activate Nek2 and Aurora-A kinases [58,59]. For these functions, I2 seems to operate through its C-terminal domain as the protein deleted in this domain (I2(1–118)) failed to interact with these kinases, excluding a role for the KGILK and RVxF motifs. Although the PfI2 sequence is 61 amino acids shorter than its human homologue, the capacity of PfI2 to bind *P. falciparum* kinases of the NIMA and Aurora families (for which active recombinant enzymes are available [60-64]) should be evaluated.

In *P. falciparum*, microarray analysis detected PfI2 mRNA in all blood parasite stages and gametocytes (data available in PlasmoDB, [41]). In this work, co-immunoprecipitation experiments with anti-PfI2 antibodies followed by Western blotting and the use of a PfPP1 affinity column clearly revealed the expression of PfI2 protein by *P. falciparum* and of its capacity to bind PfPP1. Transfection of live parasites with the tagged PfI2-GFP protein showed that its distribution is nucleocytoplasmic, like PfPP1 [28], with a strong accumulation in the nucleus, is in agreement with the localization of other I2 proteins [49]. Indeed mammalian I2 fused to GFP was localized in both the cytoplasm and the nucleus, with an active import to the latter compartment, supported by the presence of two putative nuclear localization signals [49,65,66]. In the case of PfI2, bioinformatics analysis also revealed a putative nuclear localization signal, supporting its nuclear localization. We previously reported that PfLRR1 and Pf inhibitor-3, the first identified regulatory subunits of PfPP1c, localized to the nucleus, evoking a specific role in this compartment [28,29]. The present study suggests an additional role for the PfI2 regulatory subunit of PP1c, present in the nucleus but also in the cytoplasm. Our reverse genetic studies strongly suggest a critical role for PfI2 in the erythrocytic asexual cycle *in vitro* as no parasites with a disrupted *PfI2* gene were detectable. Definition of the PfI2 role(s) during the life cycle necessitates further work, requiring the development of a powerful inducible expression system for *P. falciparum*.

The ability of PfI2 to bind and to inhibit PP1c both *in vitro* and in cellular conditions (*Xenopus* oocytes) through the two main motifs: the RVxF motif (KTISW) and the HYNE motif, together with the fact that a tight and appropriate regulation of PP1c is crucial for cellular functions, led us to explore whether derived ‘competing’ peptides from PfI2 could bind to PP1c and inhibit downstream signaling pathways. Only peptides containing the KTISW or HYNE motifs were able to bind to PfPP1c. However, the incubation of these peptides with PfPP1 or their injection into oocytes failed either to inhibit phosphatase activity or to promote GVBD respectively. However, the pre-injection of the KTISW and HYNE peptides did block the PfI2-dependent GVBD. Moreover, there was no interaction between *Xenopus* PP1 and PfI2 in extracts of oocytes pre-injected with the KTISW or HYNE peptides. This encouraged us to investigate the ability of these peptides to inhibit the growth of *P. falciparum*. To do this, the capacity of the peptides to cross membranes was first improved by including a short basic peptide, which has been shown to be highly efficient in increasing the permeability of peptides and to promote accumulation within infected red blood cells [52]. Peptides encompassing the RVxF degenerate motif R/KX_0-1_ V/I X_0-1_ F/W (KTISW or KVVRW) inhibited the growth of 3D7 *P. falciparum* strain at low micro-molar concentrations. The substitution of amino acids essential for binding with PfPP1 validated that the growth inhibition was RVxF-dependent. The difference in the observed IC50 values of KTISW and KVVRW containing peptides could be related to a higher affinity of the latter for PfPP1 and the fact that it proved able to accumulate not only in merozoites but also in parasites within infected red blood cells. Unexpectedly, the second PP1 binding peptide containing the HYNE motif, although it was found functional in oocyte model, was not active as an antiplasmodial suggesting that native PfI2 expressed by *P. falciparum* could displace the HYNE peptide. One possible explanation for the anti-parasitic activity of RVxF containing peptides is that an increase in PP1 activity due to its reduced interaction with regulators could result in uncontrolled protein dephosphorylation, leading in turn to an inhibition of parasite differentiation/growth. This implies that each competing active peptide can block its respective protein but that cross-inhibition of other partners using the same docking site cannot be excluded. These peptides might prove very useful as fundamental research tools to dissect pathways and processes controlled by PP1 in *Plasmodium falciparum*.

## Conclusion

In this study we report the molecular analysis and functional role of the inhibitor-2 regulator, a gene product that binds to and controls the activity of PfPP1. Structure-activity studies of this regulator led to the identification of binding/functional motifs of PfI2. In addition, peptides corresponding to the RVxF motif exhibit anti-plasmodial activity against blood stage parasites *in vitro*. Although, additional investigations are required to better define the interaction of competing peptides in the parasite, the proof-of-concept finding of derived peptides from regulators of PfPP1 that inhibit the binding of PfI2 to PfPP1 and, in consequence, parasite growth is an important advance. It opens new avenues for the design of novel anti-parasitic therapeutics by screening large combinatorial libraries of small compounds blocking the function of regulators of PfPP1. Interestingly, a recent study showed that the selective inhibition of GADD34, a human regulator of PP1, by guanabenz (an a2-adrenergic receptor agonist) was able to restore proteostasis and to protect stressed cells [67]. This further confirms that interfering with the interaction of PP1-regulators and/or dissociation of the complex can help to better understand the role of PfPP1 and to create new means to develop antimalarials.

## Methods

### Genome databases searches and sequences analysis

Putative Inhibitor 2 sequences were searched using BLASTp on sequences available in GenBank [68], PlasmoDB [41], ToxoDB [69], SchistoDB [70], Xenbase [71] and OrthoMCLDB [72] databases. The human I2/*Plasmodium falciparum* I2 sequences alignment was performed using the ClustalW program and was manually corrected.

### Phylogenetic analyses and secondary structure prediction

Protein sequences (listed in supplemental Additional file 4: Table S3) were aligned using the ClustalW algorithm implemented in the BioEdit v7.1 software, and manually corrected. Maximum likehood trees were built using MEGA5 [73] under the JTT + I + G model, with 100 bootstrap repetitions. of the following species: *Plasmodium falciparum , Plasmodium berghei , Plasmodium chabaudi, Plasmodium knowlesi, Plasmodium vivax, Plasmodium yoeli yoeli, Toxoplasma gondii, Arabidopsis thaliana, Homo sapiens, Mus musculus, Trypanosoma brucei, Tetrahymena thermophila, Xenopus laevis (or tropicalis), Danio rerio, Saccharomyces cerevisiae, Theileria parva, Drosophila melanogaster, Leishmania major, Oryza sativa, caenorhabditis elegans and Schistosoma mansoni.*

PfI2 secondary prediction was carried out using the PsiPred software [43] and the potential nuclear signal localization was performed using the PSORTII software [42].

### Plasmids

Plasmids pCR2.1-TOPO, pQE30, pGEX4T3, pETDuet-1, pGADT7 and pGBKT7 were purchased from Invitrogen, Qiagen, Life Sciences, Novagen and Clontech respectively. Plasmid pCAM-HA, and pCAM were kind gifts of Dr C. Doerig (Monash University, Australia). Monoclonal anti-HA and anti-Myc antibodies were purchased from Roche and Invitrogen respectively.

### Preparation of parasites

*P. falciparum* 3D7 and HB3 clones were grown according to Trager and Jensen [74], in RPMI-1640 medium supplemented with 0.5% AlbuMAX™ II (Invitrogen), 0.2 mM Hypoxanthin (CCPro) and 20 μg/ml Gentamycin (Invitrogen), in the presence of O^+^ erythrocytes. Parasites were synchronized by a double sorbitol treatment as previously described [75]. In order to isolate total RNA or proteins, parasitized erythrocytes were prepared by saponin lysis [76] and either resuspended in Trizol (Invitrogen) or in phosphate buffered saline containing EDTA-free protease inhibitor cocktail (Roche). For some experiments, infected red blood cells were purified using Percoll-sorbitol density gradients with slight modifications [77]. Soluble protein extracts were prepared from saponin-isolated parasites by suspending the pellet in lysis buffer (20 mM Tris–HCl, Nacl 150 mM pH 7.4, 0.5% Triton X-100, and protease inhibitors cocktail (Roche)) followed by 7 consecutive freezing/thawing cycles with intermediate sonication steps and subsequent centrifugation at 13 000 rpm for 30 min at 4°C. Protein concentrations in the supernatants were determined using the BCA protein assay (Pierce).

### Recombinant proteins expression and purification

Initial experiments with the wild-type *PfI2* cDNA did not allow the production of recombinant protein whatever the bacterial plasmid and the condition of expression used. In order to overcome this problem, a *PfI2* gene with optimized codons has been synthesized (eurofins, MWG operon). The sequence is presented in Additional file 5: Figure S2. This synthetic gene has been cloned in different bacterial and yeast plasmids for interaction and functional studies and used as template to obtain deleted and mutated PfI2 proteins. Briefly, the full-length coding region of PfI2WT, PfI2(1–94) and PfI2(19–144) were obtained by PCR with the primers Pr1-Pr2, Pr3-Pr4 and Pr5-Pr6 respectively (Additional file 1: Table S1) and subcloned in pQE30. For the expression of PfPP1 (primers Pr11 and Pr12), the pETDuet-1 expression system was used. The restriction sites are mentioned in Additional file 1: Table S1. Before cloning in expression vectors, all PCR products were subcloned in a pCR2.1-TOPO vector (Invitrogen) and verified by sequencing for the absence of any modification introduced by Taq polymerase.

To obtain the PfI2 mutant constructs, we performed PCR-based site-directed mutagenesis using the constructions pQE30-PfI2 or pGADT7-PfI2 as templates, the primers Pr7-Pr8 (PfI2W16A) or Pr9-Pr10 (PfI2Y103A) and using Isis Proofreading DNA polymerase (Qbiogene). The PCR conditions consisted of 1 min at 95°C followed by 16 cycles at 95°C (30 s), 55°C (1 min) and 72°C (8 min). The parental DNA plasmid was then digested with *DpnI* and an aliquot was used to transform XL10-Gold Ultracompetent cells (Stratagene). Mutated plasmids were checked by sequencing for the replacement of tryptophan 16 and tyrosine 103 by an alanine and then used for the expression of mutant PfI2 recombinant proteins or yeast two-hybrid assays.

Protein expression was carried out in the *E. coli* M15 strain for the pQE30 construct and the BL21 strain for pETDuet-1 constructs. The expression of His6-PfI2 proteins was carried out in the presence of 0.5 mM IPTG at 37°C for 2 hr. For the expression of His6-PfPP1, the culture was induced overnight at 16°C in the presence of 0.5 mM IPTG and 1 mM MnCl_2_. Cells were harvested in sonication buffer (20 mM Tris, 1% Triton X-100, lysozyme 1 mg/ml, and protease inhibitor cocktail (Roche)). His-tagged recombinant proteins were purified according to manufacturer’s instructions by Ni2+ chelation chromatography (GE Healthcare). With respect to the His6-PfI2 proteins, the extract was prepared using a 20 mM Tris HCl (pH 7.4), 150 mM Nacl, 20 mM Imidazole and 6 M guanidine buffer and loaded on a 1 ml nickel-NTA resin column (HiTrap, GE Healthcare). Washing steps were performed with a buffer containing 20 mM Tris HCl (pH 7.4), 150 mM NaCl and 20 mM imidazole. The imidazole-eluted proteins were dialyzed against 20 mM Tris pH 7.4, 150 mM NaCl. Under these conditions, the purity checked by SDS-PAGE followed by Coomassie-blue staining was >95%. His6-PfI2 protein was further subjected to peptide mass fingerprint by MALDI-TOF mass spectrometry to confirm its identity.

For antisera production, the purified His6-PfI2WT was mixed in Al(OH)_3_ (Alu-Gel-S, Serva) (100 μg per injection) and injected into rats (intra peritoneal route). Animals were boosted twice at intervals of 3 weeks with the same quantity of His6-PfI2. The sera were obtained 2 weeks after the last boost and tested for their titres and specificity by ELISA and Western-Blotting against recombinant proteins. Preimmune sera were used as negative control.

### Detection of PfI2 in *P. falciparum* erythrocytic stages

For Western blots, 60 μg/lane of *P. falciparum* soluble proteins from synchronous and asynchronous cultures were separated on a 4-12% SDS-PAGE and subsequently blotted onto nitrocellulose. For the detection of PfI2, the blots were probed with primary rat anti-PfI2 at 1:50. For co- immunoprecipitation experiments, soluble parasite extracts were incubated with anti-PfI2 polyclonal antibodies in the presence of sepharose-protein G. After several washings, the eluates were separated by SDS-PAGE (4-12%gel) and transferred to nitrocellulose. Immunoblot analysis was performed with anti-PfI2 antibodies. The detection of endogenous PfI2 in total proteins extracted from asynchronous cultures of *P. falciparum* were also carried out by using PfPP1 chromatography column. Briefly, 10 mg of total protein extracts pre-cleared on Ni-NTA sepharose beads were incubated overnight with His6-PfPP1 affinity Ni-NTA column. After washings, proteins eluted with SDS-PAGE loading buffer were migrated and blotted to nitrocellulose. The blots were probed with preimmune serum anti-PfI2 or with anti-His mAb antibodies. All secondary antibodies were purchased from Jackson ImmunoResearch laboratories. Horseradish peroxidase-labeled anti-mouse IgG (1:1000 dilution), anti-rat (1:500) were used as secondary antibodies followed by chemiluminescence detection (Santa Cruz Biotechnology).

### Localization of PfI2

For an episomal expression of PfI2-GFP, the full-length coding region of *PfI2* was amplified by PCR using the primers Pr23 and Pr24 (Additional file 1: Table S1) containing XhoI and KpnI restriction sites respectively. The PCR fragment was cloned into pCR2.1-TOPO vector (Invitrogen) and its nucleotide sequence was verified. The PCR product was then subcloned in frame with GFP into pARL vector (Kind gift of the Dr C. Sanchez Heidelberg, Germany) [47] digested with XhoI and KpnI. The plasmid carries the human *dhfr* gene for selection with WR99210 and the *PfCRT* promoter. The populations of stably transfected parasites were obtained after 6 weeks. Live parasites were analysed and images were recorded by fluorescence microscopy (Zeiss, LSM710).

### Generation of *P. falciparum* transgenic parasites

The *PfI2* disruption plasmid (pCAM-*PfI2*) was generated by inserting a PCR product corresponding to a 5′ portion from the *PfI2* sequence (720 bp) into the pCAM-BSD vector which contains a cassette conferring resistance to blasticidin. The insert was obtained using 3D7 genomic DNA as template and the oligonucleotides Pr19 and Pr20 (Additional file 1: Table S1), which contain PstI and BamHI sites respectively. Attempts to check the accessibility of *PfI2* locus were performed by transfecting wild 3D7 parasites with 3′ tagging constructs. To this end, the 3′ end of the *PfI2* sequence (695 bp, omitting the stop codon) was amplified by PCR using 3D7 genomic DNA and the primers Pr21 and Pr22 containing PstI and BamHI restriction sites respectively. The 3′ tagging plasmids were generated by inserting the PCR product into PstI and BamHI sites of the pCAM-BSD-hemagglutinin (HA). Transfections were carried out by electroporation of ring stage 3D7 parasites with 75–100 μg of plasmid DNA, according to Sidhu *et al.*[78]. To select transformed parasites, 48 h after transfection, Blasticidin (Invivogen) was added to a final concentration 2.5 μg/ml. Resistant parasites appeared after 3–4 weeks and were maintained under drug selection.

### Genotype and phenotype analysis of *P. falciparum* transfectants

To check the presence of correct constructs in transfected parasites, plasmid rescue experiments were carried out. Genomic DNA extracted (KAPA Express Extract, kapaBioSystems) from wild or transfected parasites were used to transform *E*. *coli DH5*α cells (Invitrogen). Plasmid DNA was then purified from bacterial clones and digested with PstI and BamHI.

Genotypes of *PfI2* knock-out parasites were analyzed by PCR on genomic DNA using standard procedures with the primers Pr 27 (derived from the 5′ non-translated region and not present in the construct) and Pr26 specific for the pCAM-BSD vector. Genotypes of *PfI2* knock-in were analyzed using the primer Pr19 and Pr 28 (reverse primer corresponding to HA).

### Assays for PfPP1 and effect of PfI2

The activity of PfPP1 with *p*-nitro-phenylphosphate (pNPP) was assayed as previously described [28]. To investigate the role of PfI2 recombinant proteins or PfI2/PfI3 derived peptides on His6-PfPP1 activity, different amounts of proteins were added to 1 μg of PfPP1 recombinant protein and preincubated for 30 min at 37°C before testing the PfPP1 phosphatase activity. Okadaic acid was used as control (data not show). Results are presented as mean of increase or decrease of phosphatase activity in comparison to His6-PfPP1 incubated in the reaction buffer.

### Yeast two-hybrid assays

The full length *PfPP1* was cloned into the pGBKT7 vector containing the DNA binding domain of *gal4* (Gal4-BD) and wild-type, deleted or mutated *PfI2* (*PfI2WT, PfI2(1–94), PfI2(19–144), PfI2W16A, PfI2Y103A*) into pGADT7 containing the *gal4* activation domain (Gal4-AD). The *pGBKT7-Gal4-BD-PfPP1* construct was used to transform Y187 strain and maintained on SD media without tryptophan (SD-W). The *pGADT7-Gal4-AD-PfI2* constructs were used to transform AH 109 strain and maintained on SD media lacking leucine (SD-L). Mating these two haploid strains results in the formation of diploid strain, which is viable on SD media lacking leucine and tryptophan (SD-LW). Interaction of PfPP1 with the different versions of PfI2 proteins were evaluated by their capacity to grow on selective media: SD medium lacking leucine, tryptophan and histidine (SD-LWH) and SD medium lacking leucine, tryptophan, histidine and adenine (SD-LWHA) for 4 days. Yeasts transformed with empty vector or with pGBKT7 laminine were used as controls.

### Induction of *Xenopus* oocytes germinal vesicle breakdown and co-immunoprecipitation

Preparation of *Xenopus* oocytes and microinjection experiments were performed as previously described [79]. Briefly, in each assay, 20 oocytes removed from at least two or three different animals were microinjected with His6-PfI2 (wild type, deleted or mutated) recombinant proteins or PfI2/PfI3 derived peptides. Preliminary experiments using different concentrations ranging from 40 to 120 ng per injection showed that 100 ng of PfI2 was sufficient to induce the highest rate of GVBD. Okadaic acid was used as a positive control for ability of oocytes to respond to PP inhibition (data not shown). Regarding the pre-injection experiments, deleted, mutated His6-PfI2 proteins or PfI2/PfI3 derived peptides were pre-injected (100 ng) in the oocytes 1 hour before the His6-PfI2WT injection (100 ng). GVBD was detected by the appearance of a white spot at the apex of the animal pole after 15 hours. Oocyte extracts were prepared as follow: oocytes were lysed in buffer (50 mM HEPES pH 7.4, 500 mM NaCl, 0.05% SDS, 0.5% Triton X100, 5 mM MgCl2, 1 mg/ml bovine serum albumin, 10 μg/ ml leupeptin, 10 μg/ml aprotinin, 10 μg/ml soybean trypsin inhibitor, 10 μg/ml benzamidine, 1 mM PMSF, 1 mM sodium vanadate) and centrifuged at 4°C for 15 min at 10,000 g. To detect His6-PfI2 proteins in injected extracts, electrophoresis followed by western-blot analysis performed on oocytes extracts. The membranes were developed with anti-His mAb antibody (Qiagen).

To examine the interaction of PfI2WT with XePP1, we carried out co-immunoprecipitation experiments with extracts from oocyte injected with PfI2 using anti-His mAb antibodies (1:100) (Qiagen) or anti-rabbit antibodies (1/100) (Santa Cruz Biotechnology) in the presence of sepharose-protein G. Elutes were analysed as described above using anti-PP1 antibodies (Santa-Cruz Biotechnology) (1:15 000) or by anti-His antibodies (1:10 000) and the advanced ECL detection system (Santa Cruz Biotechnology).

### Binding of PfPP1 with synthetic peptides derived from PfI2

The peptides listed in the supplementary Additional file 3: Table S2 were purchased from Genscript (USA) with a purity > 98%. All peptides were solubilized in PBS and used in an ELISA based assay as previously described [80]. Plates were coated with 50 μg/ml (100 μl) of each peptide proteins or 10 μg/ml of PfI2WT in PBS overnight at 4°C. Following washing with PBS-Tween20 0.1%, the plates were blocked with PBS containing 0.5% gelatine for 1 hour at room temperature. Coated-plates were then incubated with different concentrations of biotinylated PfPP1 which has been labelled with biotin-NHS according to the manufacturer’s instructions (Calbiochem). Incubation of biotin-PfPP1 with the different peptides or proteins was performed in PBS-Tween 0.1% at 37°C for 2 hrs. After 5 washes with PBS-Tween 0.1%, binding was detected using streptavidin-HRP. After a period incubation of 30 min and 5 washes, TMB substrate (Uptima) was added and the reaction stopped using 2 N HCl. The OD was measured on an ELISA plate reader (Dynex MRX II) at 450 nm.

### Growth inhibition assay of *P. falciparum*

Assays were carried out in 96-well plates with a starting parasitemia of 0.5% at a haematocrit of 1% using SYBR Green I [81]. The peptides were added to the culture at different concentrations ranging from 80 μM to 1.25 μM (final concentrations) in a volume of 250 μl of RPMI-AlbuMAX (0.5%) and incubated for further 72 hr to allow all stages to complete at least one cycle. Cultures were stained for 30 minutes in the dark with SYBR Green I 1X (Invitrogen) diluted in 20 mM Tris pH8.8, 138 mM NaCl, and fixed with 1% paraformaldehyde. Fixed pRBC were stored at 4°C in the dark until flow cytometry analysis.

Parasite growth was assessed by flow cytometry on a FACSCalibur (Becton-Dickinson). Infected and uninfected erythrocytes were gated on the basis of their forward scatter (FSC) and side scatter (SSC) signals. Fluorescence analysis (Green fluorescence, FL-1 filter) was performed with CellQuest software (FACScan; BD Biosciences) on a total of 100,000 acquired events. Fluorescence was observed as described by Izumiyama *et al.*[82] on a two-parameters dot plot (FL1-SSC). Fluorescence of non-infected RBC was adjusted to plot between 10^0^ and 10^1^. Results are expressed in percentage of fluorescence among total RBC.

The drug concentration resulting in 50% inhibition of parasite growth (IC50) was assessed by determining the drug concentration corresponding to 50% of the parasitaemia observed in the peptide-free control wells. The IC50 value was calculated using the ICEstimator software [83] based on a non-linear regression analysis of log-based dose–response curves. Results are presented as means ± sem.

### Analysis of peptide uptake by *P. falciparum* infected red blood cells

FITC-labeled P1 and P5 peptides were added at a final concentration of 20 μM to *3D7 P. falciparum* infected erythrocytes (10% parasitaemia). The parasite nucleus was stained using DAPI (Sigma-Aldrich). FITC-labeled P1 and P5 peptide penetration was analysed by fluorescence microscopy (Zeiss, LSM710).

### Toxicity studies

The cytotoxic effect of peptides was assessed using murine splenocytes stimulated by concanavalin A [81]. Cells isolated from BALB/c mice and washed twice in RPMI 1640 medium, were resuspended in RPMI 1640 supplemented with 1X non-essential amino acids (Invitrogen), 4 mM glutamine (Cambrex), 10% FBS, 5 μg/ml gentamycin, 50 μM β-mercaptoethanol (Merck), and 1 μg/ml concanavalin A (Sigma). Cells (2.10^5^ cells /well in 100 μl) were then seeded into 96-well flat-bottom tissue culture plates containing peptides (100 μl) serially diluted with complete culture medium. The plates were incubated for 72 h in a humidified atmosphere at 37°C and 5% CO2. 20 μl of a stock solution of resazurin (alamar Blue, AbD Serotec, Oxford UK) were then added per well (final concentration 10 μM), and the plates were further incubated at 37°C for 24 h. Optical densities were measured in a DYNEX MRX II plate reader with excitation wavelength at 570 nm and emission wavelength at 620 nm. The calculations (difference in reduction between control and treated cells) were done according to the recommendations of manufacturer (AbD Serotec). The 50% inhibiting concentration of cell proliferation (IC50) were calculated by locating the x-axis values corresponding to one-half of the absorbance values. Results are presented as means ± sem.

## Competing interests

There are no competing interests by any of the contributing authors.

## Authors’ contributions

AF and JK: Designed the study. AF, KCM, CP, GT, HK, SL and AM performed experiments. AF, KCM, CP, AM, RP, J-FB, JK analyzed data. AF, KCM, RP, J-FB, and JK wrote the paper. All authors read, contributed feedback to, and approved the final manuscript.

## Supplementary Material

Additional file 1: Table S1 List of the primers used throughout this study. Click here for file

Additional file 2: Figure S1 Nucleotide sequence of *P. falciparum* inhibitor 2. Sequence of PfI2 obtained by RT-PCR using different sets of primers to confirm the start and the stop codons. The 5^′^ and 3^′^ non -coding sequences are presented in italic. The start and stop codons are bolded. Click here for file

Additional file 3: Table S2 list of the peptides used throughout this study. Click here for file

Additional file 4: Table S3 list of the proteins present in genomic database used in the phylogenetic analysis. Click here for file

Additional file 5: Figure S2 Optimized sequence of PfI2 used throughout this study for recombinant protein expression and interaction studies in yeast. Click here for file
