# Supplementary figures and images for: Plasmodium falciparum encodes a conserved active inhibitor-2 for Protein Phosphatase type 1: perspectives for novel anti-plasmodial therapy
Source: BMC Biol. 2013 Jul 9;11:80. doi: 10.1186/1741-7007-11-80 (PMC3735429; doi:10.1186/1741-7007-11-80)

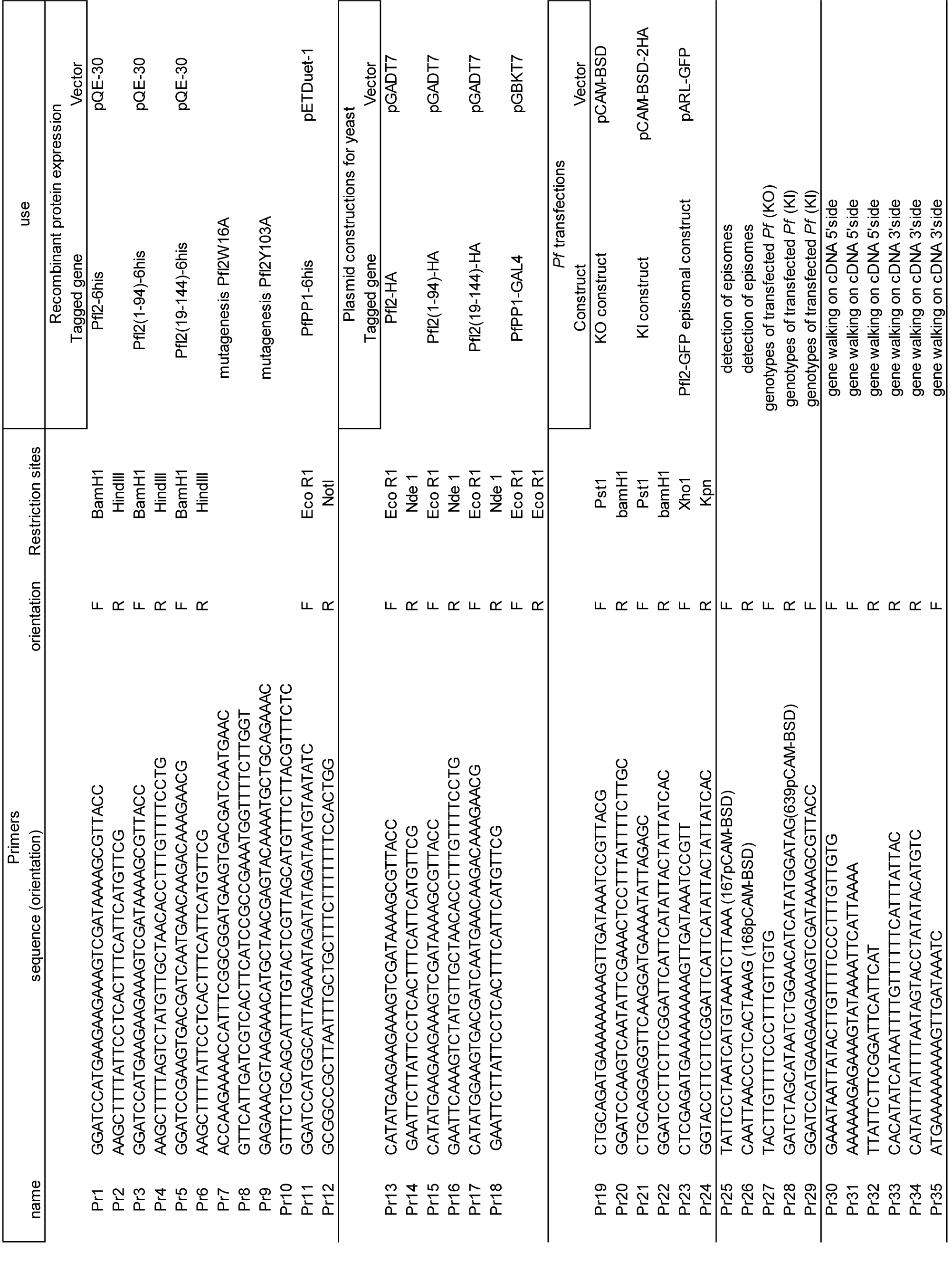

Supplement: Additional file 1: Table S1 — List of the primers used throughout this study. [file 1741-7007-11-80-S1.tiff]

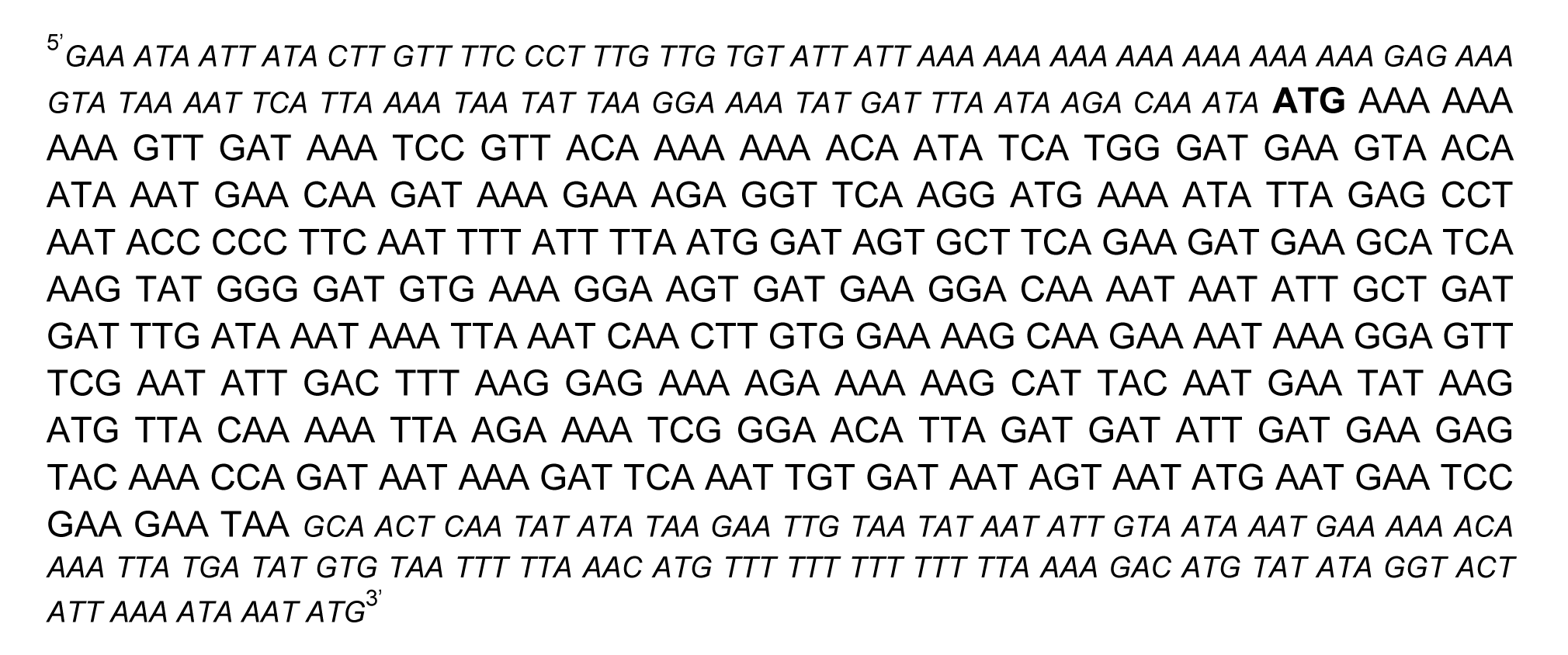

Supplement: Additional file 2: Figure S1 — Nucleotide sequence of P. falciparum inhibitor 2. Sequence of PfI2 obtained by RT-PCR using different sets of primers to confirm the start and the stop codons. The 5′ and 3′ non -coding sequences are presented in italic. The start and stop codons are bolded. [file 1741-7007-11-80-S2.tiff]

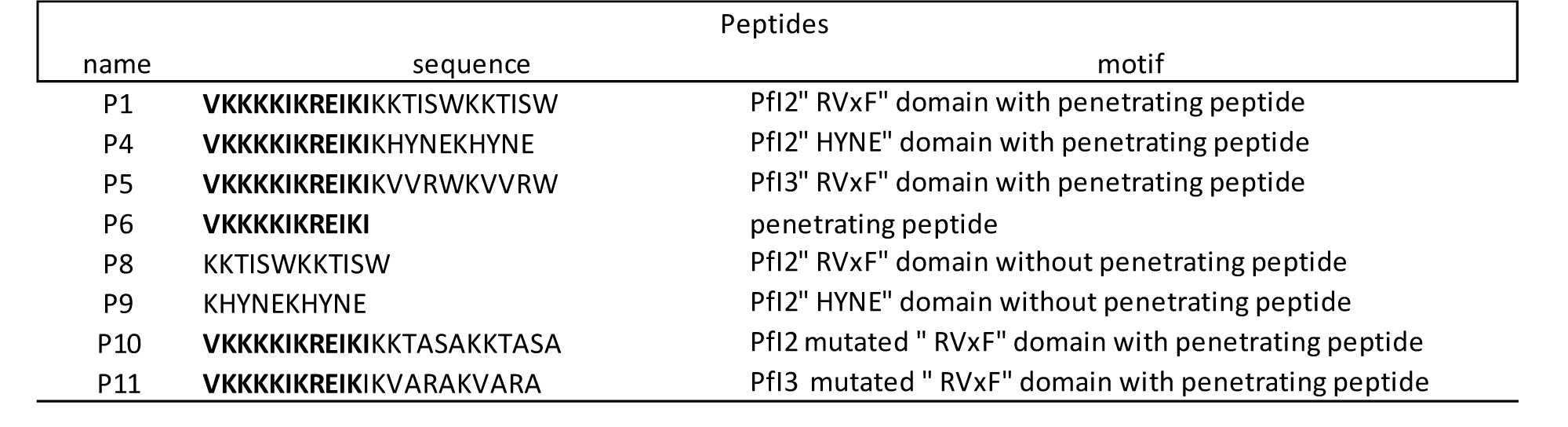

Supplement: Additional file 3: Table S2 — list of the peptides used throughout this study. [file 1741-7007-11-80-S3.tiff]

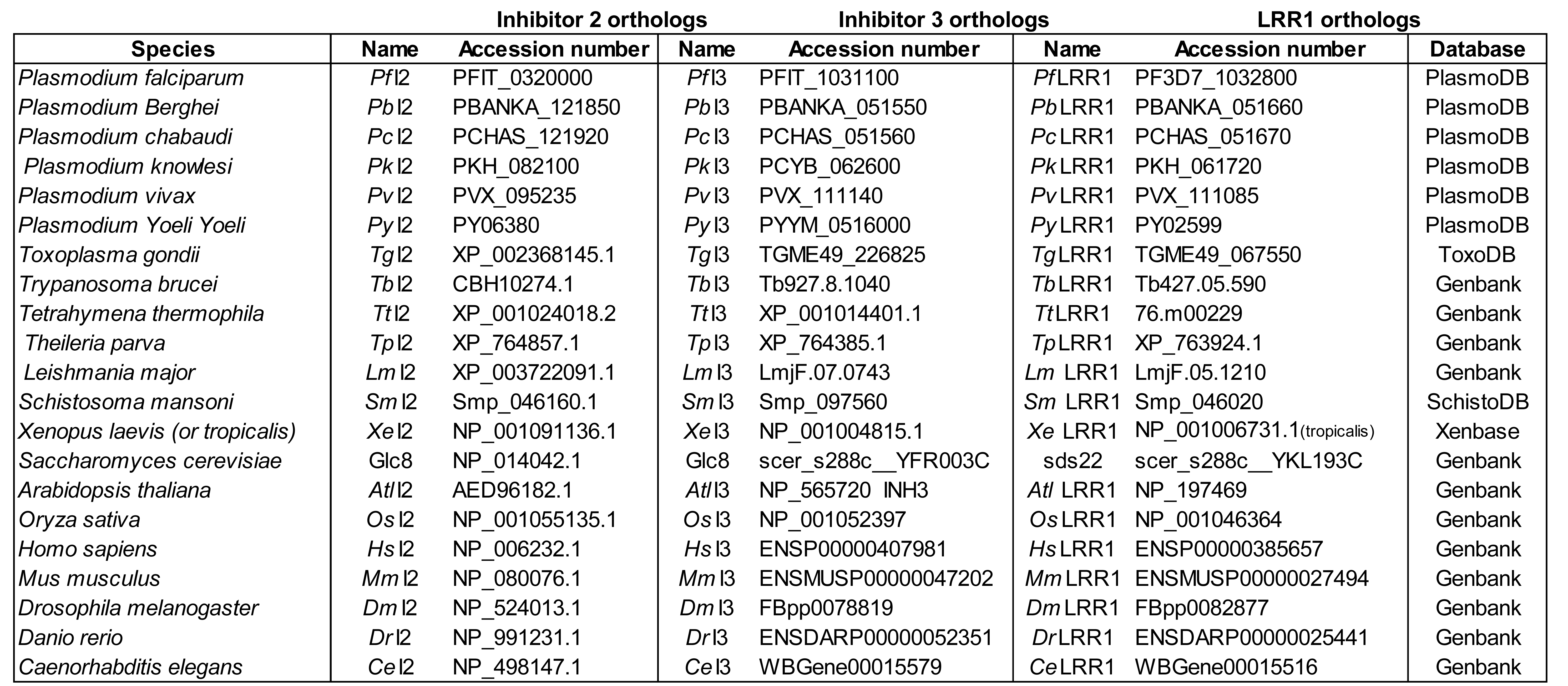

Supplement: Additional file 4: Table S3 — list of the proteins present in genomic database used in the phylogenetic analysis. [file 1741-7007-11-80-S4.tiff]

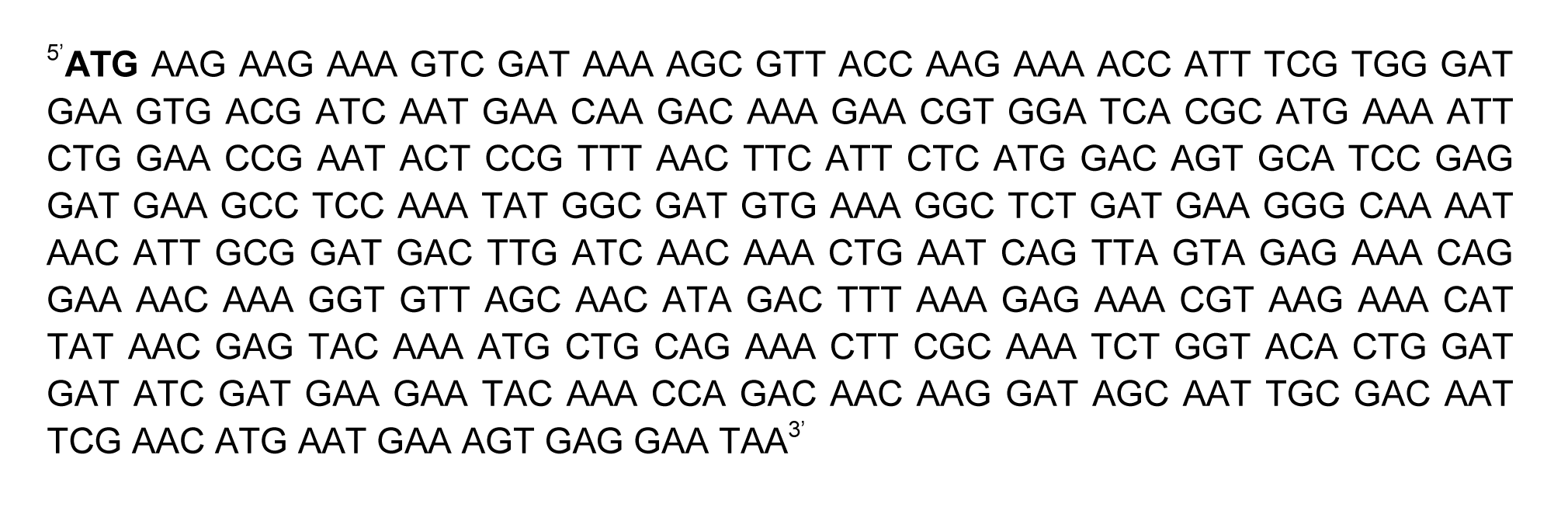

Supplement: Additional file 5: Figure S2 — Optimized sequence of PfI2 used throughout this study for recombinant protein expression and interaction studies in yeast. [file 1741-7007-11-80-S5.tiff]
